# Autistic traits specific to communication ability are associated with performance on a Mooney face detection task

**DOI:** 10.3758/s13414-024-02902-w

**Published:** 2024-05-16

**Authors:** Michael C. W. English, Murray T. Maybery, Troy A. W. Visser

**Affiliations:** School of Psychological Science, The University of Western Australia, Crawley, WA Australia

**Keywords:** Face perception, Face recognition, Neuropsychology, Autism, Autistic traits, Global processing, Face inversion

## Abstract

Difficulties in global face processing have been associated with autism. However, autism is heterogenous, and it is not known which dimensions of autistic traits are implicated in face-processing difficulties. To address this gap in knowledge, we conducted two experiments to examine how identification of Mooney face stimuli (stylized, black-and-white images of faces without details) related to the six subscales of the Comprehensive Autistic Trait Inventory in young adults. In Experiment 1, regression analyses indicated that participants with poorer communication skills had lower task sensitivity when discriminating between face-present and face-absent images, whilst other autistic traits had no unique predictive value. Experiment 2 replicated these findings and additionally showed that autistic traits were linked to a reduced face inversion effect. Taken together, these results indicate autistic traits, especially communication difficulties, are associated with reduced configural processing of face stimuli. It follows that both reduced sensitivity for identifying upright faces amongst similar-looking distractors and reduced susceptibility to face inversion effects may be linked to relatively decreased reliance on configural processing of faces in autism. This study also reinforces the need to consider the different facets of autism independently.

**Supplementary Information:**

The online version contains supplementary material available at 10.3758/s13414-024-02902-w.

## Introduction

Face processing, which includes detection, identification, and emotion recognition, has long been noted as an area of difficulty for many individuals on the autism spectrum (Sasson, 2006; Weigelt et al., 2012). One prominent account for this deficit is that holistic or global processing is both a critical component of face processing ability (Goffaux & Rossion, 2006) and the dominant processing style for most of the general population (Navon, 1977), whereas autistic individuals prefer processing visual information in a piecemeal manner that focuses on the ‘local-level’ details (Happé & Booth, 2008; Mottron et al., 2006). Whilst this local processing preference may confer advantages in certain situations (e.g., superior performance in detecting hidden ‘embedded’ shapes in larger figures; Shah & Frith, 1983), it potentially hinders the processing of configurative stimuli like faces (Gerlach & Starrfelt, 2018; Lahaie et al., 2006; López et al., 2004).

**Fig. 1:**
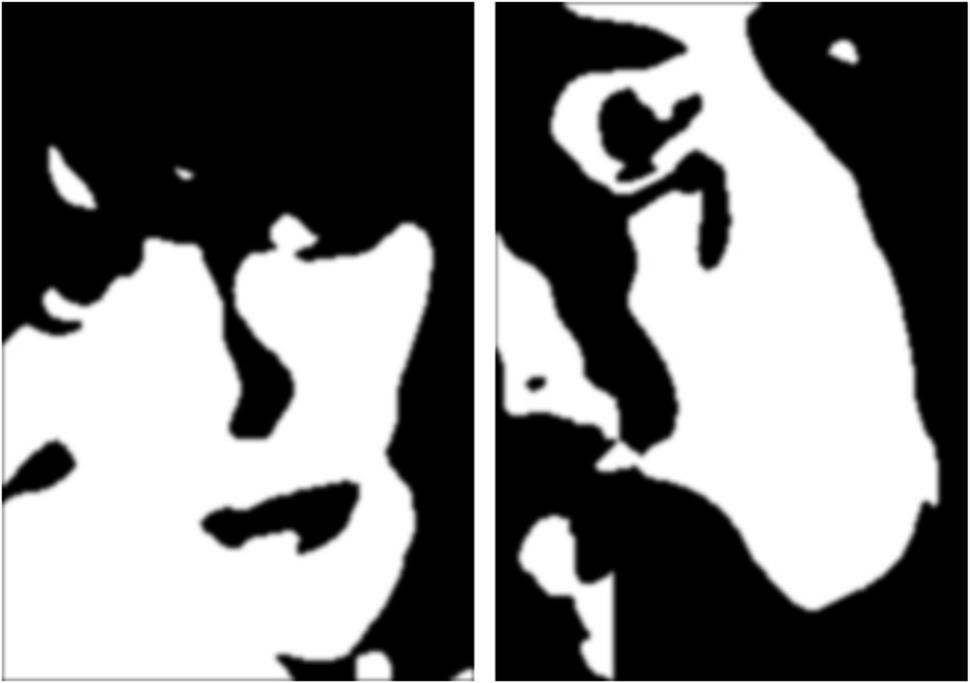
Examples of face-present (left) and face-absent (right) trial stimuli used in the experiments

Recently, investigations into this theoretical account have begun to use Mooney face visual stimuli—highly stylized black-and-white images of human faces that have had most of the details and high spatial frequency information stripped from them, leaving only the global structure (see Fig. 1 for an example; Mooney, 1956, 1957). Hypothetically, if global processing operates comparably between autistic and nonautistic individuals, there should be no group differences in accuracy on a face detection task that uses these Mooney stimuli since they have little-to-no task-relevant local-level information in them (Kanwisher et al., 1998). Conversely, if global processing limitations contribute to face processing difficulties in autism, then detection of Mooney faces should also be difficult for autistic individuals compared with their nonautistic peers.

Pursuing this line of reasoning, several studies have used Mooney face stimuli in a paradigm where participants are presented with a single brief-duration image and must report whether they saw a face or not in the image. Despite relatively small samples, three studies (Sun et al., 2012; Castelhano et al., 2018; Naumann et al., 2018) found evidence that face detection performance (accuracy and/or reaction time) was better for nonautistic compared with autistic individuals. In contrast, two other studies examining associations between Mooney face detection and subclinical autistic traits reported no correlations between face detection accuracy and measures of overall (Verhallen et al., 2014) or specific (Walker et al., 2023) autistic traits. Results of these studies are summarized in greater detail in Table 1.

**Table 1:** Summary of studies that have used Mooney face stimuli in the context of autism

Study	Participants		Mooney image stimuli	Outcome
Sun et al. (2012)	16 nonautistic, 13 autistic		Upright faces & scrambled nonfaces. 200-ms exposure.	Nonautistic faster and more accurate for faces. No difference for nonfaces.
Castelhano et al. (2018)	10 nonautistic, 10 autistic		Upright faces, inverted faces, & scrambled nonfaces. 1,000-ms exposure.	Nonautistic more accurate for inverted faces. No accuracy difference for other stimulus types, or response time difference for any stimuli.
Naumann et al. (2018)	19 nonautistic, 19 autistic		Upright faces & inverted faces. 500-ms exposure.	Nonautistic faster and more accurate across both stimulus types.
Verhallen et al. (2014)	316 university undergraduates who completed the AQ		Three-alternative forced-choice (3AFC) task (one upright face and two nonfaces per trial). Unlimited exposure.	No associations of accuracy with AQ scores. Response time not recorded.
Walker et al. (2023)		264 general population who completed the AQ	3AFC task (one upright face and two nonfaces per trial). 5,000-ms exposure. General (nonface) figure closure ability separately assessed (i.e., a task where participants try to identify visual stimuli with incomplete information).	AQ Communication^a^ scores associated with accuracy for general figure closure, but not face identification. However, indirect link found between AQ Communication and face identification through figure closure. RT not recorded.

^a^AQ Communication scores in this study refer to the Autism-Spectrum Quotient (Baron-Cohen et al., 2001) Communication factor defined by Russell-Smith et al. (2011).

AQ = Autism-Spectrum Quotient (Baron-Cohen et al., 2001).

Several methodological differences could explain the discrepant results between studies using autistic and subclinical autistic trait participants. For example, the subclinical trait studies used a different three-alternative forced-choice (3AFC) task where participants were simultaneously presented with an image of an upright Mooney face (target) and two other scrambled nonface images (distractors) and had to identify the face image. Additionally, image viewing time was much longer in the studies with subclinical autistic trait participants (5,000 ms, Walker et al., 2023; unlimited, Verhallen et al., 2014), compared with studies using autistic individuals (viewing times: 200-1000ms). Importantly, a meta-analysis on global/local processing ability suggests that autistic individuals take longer to process global structures than nonautistic individuals but are otherwise comparable in global/local processing ability (Van der Hallen et al., 2015). Thus, the longer viewing times used by Verhallen et al. (2014) and Walker et al. (2023) might have prevented detecting small differences in the ability to process faces efficiently due to differing levels of autistic traits.

A third possible explanation is that specific autistic trait dimensions are linked to Mooney face processing ability and not others. Walker et al. (2023) examined this possibility to some degree in their study, as they looked at the relationship between face detection accuracy on the 3AFC task and social skills, communication, and attention-to-details Autism-Spectrum Quotient (AQ) subscale scores as defined by Russell-Smith et al. (2011). While the authors found no direct associations between the subscales examined and face detection accuracy, an indirect relationship was reported where greater communication difficulties were linked to poorer face identification accuracy via general figure closure performance. Interestingly, this outcome differs to findings from studies examining visual processing performance using the embedded figures test (EFT; Witkin, 1971), which linked greater local processing biases with reduced social skills (Russell‑Smith et al., 2012). This suggests the aspects of autism that relate to visual processing of face and nonface stimuli are divergent.

The results of Walker et al. (2023) present tantalizing evidence that specific autistic trait dimensions could be differentially linked to face processing. However, what is needed is further work looking at more autistic trait dimensions more reliably. To this end, we administered a version of the Mooney face task similar to that used by (Sun et al., 2012) to a large, unselected sample of young adults (*N* = 335). Instead of the AQ, participants completed the Comprehensive Autistic Trait Inventory (CATI; English et al., 2021), an alternative measure of autistic traits that not only has greater reliability than the AQ but also includes trait dimensions absent from the AQ (e.g., sensory sensitivity). If some dimensions of autism are associated with face processing but not others, then only certain specific CATI subscales (corresponding to autistic trait dimensions) should be associated with face identification accuracy.

## Experiment 1

### Method

#### Participants

Participants were 335 adults (184 female, six sex not reported) with a mean age of 23.23 years (*SD* = 6.46, range: 16–42). Participants were either enrolled undergraduate students recruited through a research participation program at the University of Western Australia (*n* = 198), or members of the general population recruited through Prolific Academic (*n* = 137). Undergraduate students received partial course credit whilst the remaining participants received £1.50 for their participation.

#### Materials

##### Mooney task

Task stimuli were highly stylized black-and-white images which were previously generated by Schwiedrzik et al. (2018), who created 504 Mooney face stimuli by finding photos of faces on the internet that were shadowed and at an oblique angle, applying a gaussian blur, and maximizing the contrast, resulting in a two-tone image. Schwiedrzik and colleagues generated a further 98 scrambled-face stimuli (i.e., nonfaces) by manually rearranging segments of the face images whilst retaining the smooth edges seen in the face stimuli (see Fig. 1 for examples).We generated additional scrambled faces by creating mirrored versions of the original 98 scrambled faces (i.e., flipped along either the horizontal or vertical plane, or both planes) to total 392 scrambled nonfaces.

Prior to the current experiment, the 504 Mooney faces and 392 scrambled ‘nonfaces’ were piloted on a group of participants as a face detection task similar to the task used in the present study. As two equivalent stimulus sets were required as part of a concurrent study, the pilot participants’ task accuracy data (hits and correct rejections) were used to create two sets of images of equal difficulty with each set contained 100 face stimuli and 100 scrambled, nonface stimuli. Study participants were alternately assigned to view one of the two sets. Statistical analyses (see Results) found no effect of stimulus set assignment on task outcomes.

Stimuli were 6-cm high and 4-cm wide (following scaling; see Procedure), which equates to 4.66° x 6.72° at a viewing distance of 50 cm, and presented on a neutral grey background. Face stimuli were always oriented upright. An image of random black-and-white pixels matching the stimulus dimensions of the Mooney images was used as a mask.

**Fig. 2:**
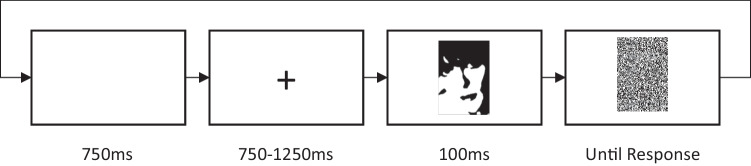
Illustration of the order and timing of events in a single trial (diagram not to scale)

Participants completed 200 trials comprising 100 face and 100 nonface images presented in random order. The timing of events for each trial is summarized in Fig. 2. Each trial began with a blank screen that was displayed for 750 ms. This was replaced with a centrally presented fixation cross that was displayed for a random duration between 750 and 1,250 ms (in multiples of the refresh rate of the display used by each participant) to prevent response anticipation. Following the presentation of the fixation cross, a Mooney image was centrally presented for 100 ms and immediately followed by the mask, which also marked the beginning of the response window. The mask remained on-screen until a response was made, although participants were instructed to respond within 2,000 ms of mask onset.

Responses were made using the ‘Z’ and ‘M’ keys on the participants keyboard, with half of the participants (randomly assigned) using the ‘Z’ key for ‘face present’ and the ‘M’ key for ‘face absent’ and the other half using the opposite response mapping. The trial ended following a response. Reminders for the response mapping were displayed in small text at the bottom of the window throughout the experiment.

##### Comprehensive Autistic Trait Inventory (CATI)

The CATI is a 42-item self-report questionnaire designed to measure traits and characteristics qualitatively like those shown by autistic individuals (English et al., 2021). It is composed of six subscales, each comprising seven items and measuring a specific trait dimension: *Social Interactions, Communication, Social Camouflage, Cognitive Rigidity, Repetitive Physical Behaviours, and Sensory Sensitivity*. Items take the form of statements to which participants indicate their level of agreement. Responses are made using a 5-point Likert scale (*strongly agree, somewhat agree, neither agree nor disagree, somewhat disagree, strongly disagree*) and are scored 1–5 (reverse-keyed responses scored accordingly). Total scale scores range from 48 to 240 and subscale scores range from 7 to 35, with higher scores indicative of greater levels of autistic traits.

#### Procedure

The experiment was administered online using the survey platform Qualtrics running in a Chromium-based browser on the participant’s own computer (tablet and mobile devices were not allowed). The Mooney face task was designed in lab.js (Henninger et al., 2019) and embedded into the Qualtrics survey. The experiment began with an instruction to maximize the browser window and to remove distractions for the duration of the 15–20 minute experiment. As participants were using their own devices, which varied in screen size and resolution, a screen calibration process was completed where participants had to resize an on-screen rectangle to match the size of a standard bank card, thus providing a common frame of reference that could be used to scale task stimuli accordingly.

Next, participants completed the Mooney face task. Participants were instructed that they would be viewing a series of images and their task was to decide whether a face was present or absent in each sequence. Several example images demonstrated the types of stimuli that would be presented in the task. Following the task, participants provided some basic demographic information and completed the CATI, at which point the experiment finished and participants were provided with debrief material.

### Results

Initial indices of task performance were accuracy, defined as the proportion of trials where participants correctly identified or rejected the presence of a face in each trial, and median reaction time (RT) for correct trials. Participants were excluded from analyses if their overall accuracy was below 50% (i.e., chance level). Nine participants were removed for below-chance accuracy leaving 326 participants (176 female, six sex not given) for subsequent analyses. Additionally, data from trials with RTs <200ms or >2000ms was excluded from analysis (mean trials excluded per participant = 5.41%).

Overall task performance, and performance for face and scrambled images separately, are summarized in Table 2. Overall accuracy and RT were weakly correlated (*r* = −.16, *p* = .003), indicating a small speed–accuracy trade-off. Paired-samples *t* tests revealed significant differences between the trial types for both accuracy and RT (both *p*s < .001, both *d*s > .20), with less accurate but faster performance found for face images compared with scrambled (nonface) images.

**Table 2:** Accuracy and reaction time (RT) for correct trials for the different images (Experiment 1)

	All images	Face images	Scrambled images
Accuracy	75.47% (8.83%)	73.01% (15.15%)	77.89% (14.82%)
RT (correct trials)	535 ms (91 ms)	517 ms (93 ms)	564 ms (113 ms)

Task accuracy was then used to compute sensitivity, *d′*, with the formula *Z [P *_*hit*_*] − Z [P *_*false alarm*_*]*, where higher scores indicate greater ability to discriminate face present and face absent stimuli. Response bias, *c*, was also calculated, using the formula −*[ Z [P *_*hit*_*] + Z [P *_*false alarm*_*] ] / 2*, where negative *c* suggests a bias towards reporting ‘face present’ and positive *c* suggests a bias towards reporting ‘face absent’. Correlations were performed between the performance measures (sensitivity and bias scores), and CATI total scale and subscale scores, which are summarized in Table 3. Between the CATI subscales, all correlations were statistically significant (*p* ≤ .001),ranged *r* = .20 to .52, and had variance inflation factors (VIF) ranging 1.39 to 1.85, indicating an absence of multicollinearity (Kim, 2019).

**Table 3:** Pearson correlations of CATI total score and subscale scores with Mooney task performance measures from Experiment 1

	Sensitivity (*d’*)	Bias (c)
Total scale	−.133*	−.017
Social interactions	.049	.001
Communication	−.292***	.029
Social camouflage	−.054	−.015
Repetitive behaviours	−.089	−.116*
Cognitive rigidity	−.069	.060
Sensory sensitivity	−.157**	−.019

*p < .05. **p < .01. ***p < .001.

Regarding sensitivity, whilst there was a small negative correlation with CATI total scale scores, indicating that poorer task sensitivity was associated with greater autistic trait levels, this effect appeared to be driven primarily by the Communication and Sensory Sensitivity subscales, which showed relatively larger, and statistically significant, negative correlations with task sensitivity. Response bias was also significantly correlated with Repetitive Behaviours scores, suggesting that higher levels of the trait were associated with a response bias favouring ‘face present’ responses.

To extend the results of the correlation analyses on task sensitivity, a follow-up linear regression analysis was conducted to determine the unique contributions of the autistic trait dimensions that significantly correlated with task sensitivity. The subscales were entered into a linear regression model as independent variables with sensitivity *d′* as the outcome measure. To enable control of demographic factors, age, sex (male, female; six participants who did not report sex were excluded from this analysis) and recruitment group (undergraduate, Prolific Academic) were entered into the regression model in an initial step (Model 1) before entering the subscales in a second step (Model 2). The results of the regression analyses are summarized in Table 4.

**Table 4:** Model statistics and standardized coefficients from stepwise linear regressions from Experiment 1 predicting Mooney face detection performance (sensitivity) using age, sex, and recruitment group (Model 1) and Communication and Sensory Sensitivity subscale scores from the Comprehensive Autistic Trait Inventory (Model 2) as independent variables

				95% confidence interval	
	*t*	*p*	β	Lower	Upper
Model 1: *F*(3, 316) = 2.21, *R*^2^ = .02, *p* = .09					
Constant	−0.438	.662			
Age	1.567	.118	0.113	−0.029	0.254
Sex	−1.237	.217	−0.074	−0.193	0.044
Recruitment group	−0.241	.810	−0.018	−0.160	0.125
Model 2: *F*(5, 314) = 7.57,* R*^2^ = .11*, p* < .001; *ΔR*^2^ = .09, *p* < .001					
Constant	2.048	.041			
Age	1.977	.049	0.136	0.001	0.272
Sex	−1.566	.118	−0.093	−0.209	0.024
Recruitment group	−0.111	.911	−0.008	−0.145	0.129
Communication	−4.909	< .001	−0.294	−0.412	−0.176
Sensory sensitivity	−0.149	.881	−0.009	−0.127	0.109

For Model 1, which tested the influence of the three demographic factors, neither the overall model nor any individual factor was statistically significant, suggesting task sensitivity was not influenced by these factors. However, Model 2, which added the Communication and Sensory Sensitivity CATI subscales to the initial model, was both statistically significant and provided a significant improvement over the initial model, with Communication scores the standout significant predictor where greater communication difficulties were associated with reduced face detection ability. Though Sensory Sensitivity was negatively associated with sensitivity in the initial correlations, this relationship was not statistically significant within the context of the regression. Of minor note, age was a significant predictor in Model 2, where greater age was associated with increased sensitivity.

Finally, a similar regression analysis was conducted to examine response biases with task bias (*c′*) as the dependent variable, the three demographic variables entered in Model 1, and Repetitive Behaviours added in Model 2. Neither model was statistically significant (Model 1: *p* = .47, *R*^2^ < .01; Model 2: *p* = .26, *R*^2^ = .02), Model 2 did not significantly improve upon Model 1(*ΔR*^2^ = .01, *p* < .10), nor were any individual predictors significant (see Supplementary Material for full analyses).

### Discussion

We recruited a large, unselected group of adults who completed the CATI, a new measure of autistic traits that spans six different dimensions, and a face detection task using Mooney face stimuli thought to preferentially recruit global processing. While correlation analyses indicated associations with the *Communication*, and *Sensory Sensitivity* subscales, linear regression revealed that communication difficulties alone accounted for unique variance in predicted greater difficulty in identifying the presence or absence of faces in the Mooney stimuli. In short, these results indicate that traits and behaviours relating to communication ability are uniquely related to the rapid detection of faces, and that other autistic related traits have a minimal or nonunique role to play in this capability.

The negative association between Communication traits and face detection ability was somewhat surprising. Previous work using the traditional Embedded Figures Test (EFT; Witkin, 1971) and Leuven Embedded Figures (de‑Wit et al., 2017) suggests that elevated scores on the social trait dimension of the AQ are associated with superior EFT performance (English et al., 2024; Russell‑Smith et al., 2012) indicating a preference for a local processing style and/or difficulty with global processing. However, both studies examined the impact of social difficulties in their samples but did not examine communication-related traits. This is an important point, because while the two trait dimensions measure different aspects of social functioning, the strength of the correlation between them (e.g. AQ: *r* = .45; CATI: *r* = .45; English et al., 2020, 2021) is substantial. Thus, it is possible that common variance between the two traits might be responsible for the associations between social difficulty and EFT performance. Put differently, past associations found between social traits and EFT performance may actually reflect communication difficulties, bringing our own findings in line with this previous literature.

Experiment 1 provided preliminary evidence that a difficulty in face detection extends to individuals with elevated scores on particular dimensions of autistic traits. Because this was demonstrated using Mooney faces for which high spatial frequency information is removed, the conjecture is that restricted utilization of global or configural information may underpin the reduced sensitivity in face detection. But the possibility remains that the processing of individual components of the Mooney faces could be implicated in performance differences, and so it would be advantageous to investigate more directly whether limited processing of configural information is central to reduced face detection sensitivity associated with autistic traits. One effect identified directly with configural processing is the face inversion effect (FIE). The FIE is the relatively reliable finding that faces that have been inverted (i.e., rotated 180°) are much harder to identify and recognize compared with their upright counterparts (Diamond & Carey, 1986; Farah et al., 1995). Critically, such effects are readily apparent for face stimuli, but attenuated or absent for other types of stimuli. (e.g., landscapes, houses; Albonico et al., 2018; Diamond & Carey, 1986). It is suggested that the mechanism underlying the typically rapid recognition that a visual stimulus is a face depends on first-order relational information—that is, spatial relations of face features that form a prototypical face (i.e., eyes, nose, and mouth appearing in descending arrangement; Diamond & Carey, 1986). Alterations to this configuration such that an image no longer appears like a prototypical upright face disrupts the typically efficient face identification processes, resulting in poorer performance (i.e., reduced accuracy or elongated response times). The FIE is typically operationalized as the difference in performance on a given task for upright versus inverted faces.

There is some precedence to expect that autistic traits may be linked to variations in the manifestation of the FIE. A recent meta-analysis (Griffin et al., 2023) suggests the FIE is reduced in autistic individuals, implying they process upright and inverted faces more similarly than do nonautistic individuals and, thus, depend less on configural mechanisms specialized for face processing (McPartland et al., 2004; Webb et al., 2012). However, the outcomes of the meta-analysis were based on studies that used a range of types of face stimuli and not just Mooney faces. Naumann et al. (2018) presented upright and inverted Mooney faces to autistic and nonautistic individuals, finding a FIE of similar magnitude for the two groups of participants for both task accuracy and response time. Castelhano et al. (2018) indicated a complementary pattern, with a 22% and 27% decrease in task accuracy respectively for nonautistic and autistic individuals for inverted versus upright Mooney faces (though the authors did not statistically test this comparison, and the effect was likely nonsignificant due to small *n* and large *SE*).

To address the preceding discussion, we conducted a follow-up experiment that closely followed the design of Experiment 1, but introduced additional inverted faces to the stimulus set, with two aims: (1) to test for replication of the effects reported in Experiment 1, and (2), importantly, investigate whether the magnitude of the FIE is reduced as a function of any of the autistic trait dimensions, consistent with diminished reliance on configural processing in the detection of upright faces. We hypothesized that:

- Following from Experiment 1, higher reported levels of communication difficulties would be associated with reduced sensitivity in distinguishing upright Mooney faces from scrambled nonface images.
- If diminished reliance on configural processing contributes to face processing difficulties associated with autistic traits, then the magnitude of the FIE should be smaller for individuals with higher levels of autistic traits, with this relationship most likely for the communication subscale of the CATI.

## Experiment 2

Experiment 2 employed a modified version of the paradigm used in Experiment 1, with the addition of inverted (i.e., upside-down) face images. With this addition, we can also report on the magnitude of the face inversion effect (i.e., the performance cost for recognizing inverted faces compared with upright faces) as well as sensitivity and bias.

### Method

#### Participants

Participants were 309 adults (230 female, two sex not reported) with a mean age of 20.56 years (*SD* = 4.28, range: 18–42). Participants were enrolled undergraduate students recruited through a research participation program at the University of Western Australia and received partial course credit for their participation.

#### Materials and procedure

The experimental design was identical to that of Experiment 1, except for the following differences. First, all participants completed the experiment using standardized hardware in a university computer laboratory—specifically, 24-in. Dell P2419H monitors operating at 1,920 × 1,080 resolution. Second, inverted (i.e., upside-down) copies were made of all the image stimuli presented to participants in Experiment 1. Crucially, this created new, inverted versions of the face images, and doubled the total number of test stimuli from 400 images to 800 images. Previously, the authors of the Mooney stimuli had verified that inverted orientations successfully reduced accuracy and increased reaction times, consistent with a face inversion effect (Schwiedrzik et al., 2018). Third, instead of participants being presented with one of two counterbalanced sets of images, all participants viewed all 800 images. A break was presented every 100 trials and participants were encouraged to take as much time as needed before proceeding with the next set of trials.

The addition of inverted faces to the stimulus set involved a necessary change in trial type ratios in order to maintain a 50:50 response ratio for face present/absent responses across the experiment. While in Experiment 1, the ratios were a simple 50:50 upright-face/scrambled-nonface presentation, Experiment 2 used a 25:25:50 upright-face/inverted-face/scrambled-nonface presentation. All other aspects of the experiment, including response keys and stimulus timings, were identical to Experiment 1.

### Results

Using the same outlier criteria as Experiment 1 resulted in the removal of one person (male) from subsequent analyses for having overall task accuracy below chancel level (i.e., 50%). Three other participants (one male) reported substantial inattentiveness or instruction noncompliance and were also removed from further analysis. Mean number of trials per participant excluded for having RTs <200 ms or >2,000 ms was 4.06%.

General task performance, and individual performance for upright and inverted face images, and scrambled images, are summarized in Table 5. Accuracy and RT were correlated (*r* = .19, *p* < .001), indicative of a speed–accuracy trade-off. Paired-samples *t* tests revealed that images of upright faces were correctly identified significantly more often than inverted faces or scrambled images (both *p*s < .02, both *d*s > .14). For correct trials, images of upright faces were also identified faster compared with inverted faces or scrambled images (both *p*s < .001, both *d*s > .33).

**Table 5:** Accuracy and reaction time (RT) for correct trials for the different images from Experiment 2 (standard deviation in parentheses)

	All images	Upright face images	Inverted face images	Scrambled images
Accuracy	65.73% (5.16%)	74.23% (13.77%)	46.98% (17.37%)	70.84% (14.21%)
RT (correct trials)	503 ms (73 ms)	489 ms (83 ms)	531 ms (102 ms)	518 ms (86 ms)

Accuracy on upright face images and scrambled (nonface) images was used to calculate sensitivity, *d′*, and response bias, *c*, following the procedure outlined in Experiment 1. Additional sensitivity and response bias indices for discriminating between inverted and scrambled nonface images were also calculated based on the same procedure. Finally, classical face inversion effects were calculated using the differences in performance measures for upright and inverted faces where accuracy, *[P *_*hit—upright faces*_*] − [P *_*hit—inverted faces*_*]*, and reaction time, *[RT *_*inverted faces*_*] − [RT *_*upright faces*_*]*, difference scores were computed such that higher values were indicative of greater negative impact of the face inversion. Correlations were performed between the performance measures, and CATI total scale and subscale scores, which are summarized in Table 6. Within the CATI subscales, all correlations were statistically significant (*p* ≤ .001),ranged *r* = .26 to .58, and VIF’s ranged 1.51 to 1.90, indicating an absence of multicollinearity (Kim, 2019).

**Table 6:** Correlations of CATI total score and subscale scores with Mooney task performance measures from Experiment 2

	Upright face vs scrambled (nonface)		Inverted face vs scrambled (nonface)		Upright vs inverted face (face inversion effect)	
	Sensitivity	Bias	Sensitivity	Bias	Accuracy	Reaction time
Total scale	−.076	−.023	−.038	−.048	−.068	−.121*
Social interactions	.007	−.066	.057	−.075	−.060	−.046
Communication	−.126*	−.009	−.077	−.048	−.091	−.116*
Social camouflage	−.025	.015	−.011	.005	−.017	−.068
Repetitive behaviours	−.054	−.053	−.005	−.075	−.077	−.127*
Cognitive rigidity	−.097	.011	−.101	−.008	−.030	−.069
Sensory sensitivity	−.063	.009	−.051	−.007	−.031	−.110

*p < .05.

#### Task sensitivity for discriminating between upright face and nonface images

In Experiment 1, we observed a strong correlation between task sensitivity and the Communication subscale, and a weaker correlation between task sensitivity and the Sensory Sensitivity subscale. In Experiment 2, the only significant correlation found was between sensitivity for upright faces versus scrambled nonfaces and scores on the ***Communication* s**ubscale. As was found in Experiment 1, reports of greater communication difficulties were associated with lower sensitivity in detecting the upright faces.

As was done in Experiment 1, we followed up this correlation with a linear regression analysis to examine the unique effect that Communication traits had on task sensitivity. The regression had task sensitivity for upright versus nonfaces as the predictor variable, the two demographic variables as predictors in Model 1 (unlike Experiment 1, a ‘recruitment group’ variable is absent as all participants were sourced from an undergraduate population), and Communication scores as an additional predictor in Model 2.

The results, summarized in Table 7, showed that Model 1 was not statistically significant, indicating that the demographics factors did not influence task sensitivity. In contrast, Model 2 approached statistical significance (*p* = .07), was a significant improvement over Model 1 (*p* = .03), and the Communication subscale was again predictive of task sensitivity, where greater communication difficulties were associated with poorer task sensitivity.

**Table 7:** Model statistics and standardized coefficients from stepwise linear regressions in Experiment 2 predicting participant sensitivity (*d′*) for discriminating upright Mooney face images from scrambled, nonface images using sex and age (Step 1) and Communication subscale scores from the Comprehensive Autistic Trait Inventory (Step 2) as predictors

				95% confidence interval	
	*t*	*p*	β	Lower	Uppe**r**
Model 1: *F*(2, 300) = 1.07, *R*^*2*^ < .01, *p* = .35					
Constant	−0.890	.374			
Age	1.237	.217	0.071	−0.042	0.184
Sex	−0.806	.421	−0.046	−0.160	0.067
Model 2: *F*(3, 299) = 2.35,* R*^2^ = .02*, p* = .07; *ΔR*^2^ = .02, *p* = .03					
Constant	0.111	.912			
Age	1.508	.133	0.087	−0.027	0.200
Sex	−0.798	.426	−0.046	−0.158	0.067
Communication	−2.212	.028	−0.127	−0.241	−0.014

#### Task sensitivity for discriminating between inverted face and nonface images

As illustrated in Table 6, neither total-scale CATI scores nor any subscale correlated with task sensitivity for inverted faces. Similarly, response bias was not associated with autistic traits, indicating that participants responded to inverted and nonface images similarly regardless of individual variations in autistic traits.

#### Autistic traits and discriminating between upright and inverted faces (i.e., face inversion effect)

The FIE as measured using task accuracy differences (i.e., *[P *_*hit—upright faces*_*] − [P *_*hit—inverted faces*_*]*) showed no significant correlations with autistic traits (see Table 6). However, the FIE as measured using reaction time differences (i.e., *[RT *_*inverted faces*_*] − [RT *_*upright faces*_*]*) showed several significant correlations. First, CATI total scale scores were significantly correlated with the FIE _RT_ measure, such that greater levels of autistic traits were associated with reduced inversion effects.

Examination of the subscales suggest that the association between the FIE _RT_ measure and autistic traits is driven by the Communication, and Repetitive Behaviours subscales, with Sensory Sensitivity approaching statistical significance (*p* = .055). As before, the subscales were examined for unique effects by using a linear regression analysis with RT FIE as the dependent variable, the two demographic factors included as predictors in Model 1 (two participants who did not report sex were excluded), and the Communication, Repetitive Behaviours, and Sensory Sensitivity subscales added in Model 2. The results of the regression analyses are summarized in Table 8.

**Table 8:** Model statistics and standardized coefficients from stepwise linear regressions from Experiment 2 predicting the Mooney face inversion effect for RT (FIE _RT_) using sex and age (Model 1), and Communication, Repetitive Behaviours, and Sensory Sensitivity subscale scores from the Comprehensive Autistic Trait Inventory (Model 2) as predictors

				95% confidence interval	
	*t*	*p*	β	Lower	Upper
Model 1: *F*(2, 300) = 2.23, *R*^2^ = .01, *p* = .11					
Constant	0.815	.416			
Age	2.097	.037	0.120	0.007	0.233
Sex	0.223	.823	0.013	−0.100	0.126
Model 2: *F*(5, 297) = 2.40,* R*^2^ = .04*, p* = .04; *ΔR*^2^ = .02, *p* = .06					
Constant	1.919	.056			
Age	2.378	.018	0.142	0.024	0.259
Sex	0.499	.618	0.030	−0.088	0.148
Communication	−1.235	.218	−0.079	−0.204	0.047
Repetitive behaviour	−0.487	.626	−0.035	−0.177	0.107
Sensory sensitivity	−1.101	.272	−0.084	−0.234	0.066

Model 1 was not statistically significant. While the addition of the CATI subscales to Model 2 resulted in statistically significant overall model, the *ΔR*^2^ only approached statistical significance, indicating that the inclusion of the subscales did not create a meaningful improvement. Given correlation analyses had shown that total-scale CATI scores, and several subscales were associated with FIE _RT_, the absence of subscale effects in the regression analyses might indicate that variations in the FIE are less attributable to specific autistic trait dimensions as assessed by the CATI, and are better explained by common aspects of autism, as exemplified in the total-scale CATI score. Finally, age appeared to play a small role in predicting the size of FIE _RT_, where increases in age were linked to larger FIE _RT_.

### Discussion

Experiment 2 attempted to replicate the findings of Experiment 1 regarding task sensitivity, whilst expanding to additionally explore the potential for distinctive face inversion effects (FIE) that might indicate whether autistic trait linked difficulties in discriminating face and nonface images in the Mooney face task are due to face-specific mechanism or not. Regarding task sensitivity for upright faces, similar effects were found to those reported in Experiment 1 where greater communication difficulties were linked to reduced task sensitivity. In contrast, sensitivity for inverted faces (versus scrambled, nonface images) showed no associations with any autistic trait dimension.

Supporting this outcome, several correlations were found between autistic traits and the FIE as calculated using response times (RT). Total-scale CATI scores were linked to a reduction in the FIE _RT_, in line with findings from a recent meta-analysis of FIE and autism (Griffin et al., 2023). While traits associated with communication difficulties and repetitive behaviours initially appeared to be driving this effect, a regression analysis suggested that unique trait effects were minimal. In other words, aspects of autism not linked to any specific trait appear to underlie the attenuation in FIE.

An interpretation of these findings is that configural processing of face features is weaker with increasing autistic traits thus resulting in a smaller FIE. Whilst a reduced reliance on configural processing of faces may reduce the negative impact of face inversion in autism, a trade-off appears to be relatively reduced sensitivity to discriminating upright faces from images of similar-looking images without faces. However, some visual processes must differ between the recognition of upright and inverted faces given Communication traits have a specific role in the identification of upright, but not inverted, faces.

It is noteworthy that the correlations between the Communication subscale and task sensitivity for upright faces was weaker in Experiment 2 compared with Experiment 1. Whilst the task parameters across the two experiments were highly similar, it is possible that the change in the ratio of specific trial types with the addition of inverted faces altered stimulus expectancy and response patterns in a manner that weakened the relationship.

## General discussion

Efficient and accurate processing of faces is understood to closely relate to the processing of the global structure of face stimuli (Goffaux & Rossion, 2006). Consequently, difficulty in face processing may be linked to a preference for local details in such stimuli. Previous work using small comparison groups of autistic and nonautistic individuals suggests that detection of Mooney faces, highly stylized black-and-white images that omit local details while retaining global structure (CITE), is slower and less accurate in autistic individuals.

Two large studies have attempted to extend this work to the general population by measuring autistic traits in unselected samples of participants and examining performance on a variation of the Mooney task (Verhallen et al., 2014; Walker et al. 2023). Though these studies found no direct associations between autistic traits and Mooney task performance, methodological differences studies may have influenced outcomes such that compatible results were not found. Consequently, it is not clear based on previous literature as to whether variation in autistic traits, either general or specific dimensions, is associated with Mooney task performance. A key aim of the present study was to determine whether specific autistic traits dimensions are related to the ability to detect Mooney faces.

An additional concern affecting all prior studies other than Walker et al. (2023) is the extent to which differences in performance on the Mooney task is due to difficulties in visual processing that are face-specific versus broader global processing. This issue was touched upon in the study by Walker et al. (2023), who administered an additional measure of general global processing ability (i.e., figure closure). While the authors found that there was an association between figure closure and the Communication subscale of the AQ, no links between autistic traits and Mooney face performance were found.

We addressed the primary concerns of the trait-based studies (namely, the long viewing times that may have affected task difficulty and thus perceptual demands) across two experiments that examined the association between autistic traits and Mooney face task performance. In both Experiment 1 and Experiment 2, we found evidence of a negative association between the Communication subscale of the CATI and Mooney task sensitivity such that greater communication difficulties were associated with poorer task performance. Experiment 2 added inverted face stimuli to the study design. This allowed us to determine if face configuration played a role in face detection differences, or if other visuospatial processing factors were responsible, and two indices were calculated to explore these possibilities.

Unlike task sensitivity for upright faces, sensitivity to inverted faces (i.e., discriminating inverted face from scrambled nonface images) showed no association with autistic traits, providing initial evidence that upright and inverted faces interacted with autistic traits differently. Supporting this view, the face inversion effect calculated from reaction times (FIE _RT_) was negatively correlated with CATI total-scale scores. indicating that individuals with greater levels of autistic traits were less impacted by the inversion of the Mooney faces in terms of response speed whilst maintaining similar accuracy levels. Combined, these outcomes suggest that face-specific processing differences might underlie the declines in Mooney task accuracy seen in autism. Critically, it appears that traits relating to communication ability have a relatively greater role to play in face processing as opposed to other autistic trait dimensions.

An interesting possibility that can be drawn from this outcome then is that it appears that autism is less negatively impacted by the apparent face processing trade-off observed for neurotypical individuals. That is, while individuals with fewer autistic traits showed greater sensitivity (and thus processing efficiency), for detecting faces amongst similar nonface images, they also showed greater disruptions for identifying inverted face images. Presumably, the processing efficiency for upright faces is highly specific for such individuals, whilst those with greater levels of autistic traits may display less orientation-specific processes than enables greater sensitivity to identifying inverted faces.

Our results also reinforce the need to treat autism and autistic traits as heterogenous and complex features with separable dimensions. In Experiment 1, this is demonstrated by the absence of a correlation between total-scale CATI scores and task sensitivity, replicating findings by Verhallen et al. (2014), but the detection of a significant association between task sensitivity and Communication traits. It is possible that a deeper analysis of the Verhallen et al. (2014) data using AQ subscale scores (e.g., the Communication subscale as identified by Russell‑Smith et al., 2011) may have revealed a pattern compatible with our Experiment 1 outcomes. In Experiment 2, while a general measure of autistic traits was negatively associated with FIE _RT_, further examination suggested that specific autistic trait dimensions may have contributed to this effect more so than others.

It is important to note that this study examined simple face detection (i.e., participants made simple face-present and face-absent responses) whereas many other face-related studies have examined the processing of identity and emotion, among other things. Furthermore, participants were explicitly searching for faces, which may have led to greater priming of face-specific configurations when viewing stimuli. Additional work is required to determine if the pattern of results reported in the present study is found in these other areas or if different patterns (and thus, different underlying processes) are present. It also remains to be seen whether detection of global face structure differs on the basis of particular traits or characteristics within clinically diagnosed autistic individuals. It is also unclear whether similar differences can be found for other “Mooney-fied” classes of stimuli such as objects (Van de Cruys et al., 2017).

A limitation of the present study is that the link between Mooney task sensitivity and the Communication subscale found in Experiment 1 was not robustly replicated for task sensitivity to upright faces in Experiment 2, with a relatively weaker association found instead. One possibility for this change in effect strength is that the addition of inverted faces altered the overall proportion of upright faces presented to participants (50% in Experiment 1, 25% in Experiment 2) may have had an unintended effect on how participants made classifications on trials with uncertainty. This may be seen in the summary statistics for each experiment. For example, while accuracy for (upright) face images was comparable between the two experiments (73% and 74%), the difference in accuracy for scrambled nonface images was relatively larger (78% in Experiment 1, 71% in Experiment 2), perhaps due to the added difficulty of distinguishing scrambled nonface and inverted face images.

In summary, the present study reveals an association between specific autistic trait dimensions and the ability to detect Mooney faces. This outcome provides an avenue for understanding previous conflicting results for clinically-diagnosed autistic individuals and nondiagnosed individuals varying in autistic traits in which specific autistic dimensions were not examined. The study also serves to reinforce the need to consider different autistic trait or symptom dimensions when studying autistic-related behaviours as complex relationships between traits or symptoms and behaviours may be missed or misattributed when autism is over-simplified into a homogenous condition.

## Supplementary Information

Below is the link to the electronic supplementary material.

Supplementary file1 (DOCX 17 KB)

## Data Availability

The data used to generate the analyses in this paper are available from the corresponding author upon reasonable request.
