# Supplementary material for: Autistic traits specific to communication ability are associated with performance on a Mooney face detection task
Source: Atten Percept Psychophys. 2024 May 16;86(7):2504–16. doi: 10.3758/s13414-024-02902-w (PMC11480180; doi:10.3758/s13414-024-02902-w)
Supplement: Supplementary file 1 — Supplementary file1 (DOCX 17 KB) [file 13414_2024_2902_MOESM1_ESM.docx]

# Supplementary Material for: Autistic traits specific to communication ability are associated with performance on a Mooney face detection task

Model statistics and standardised coefficients from stepwise linear regressions in Experiment 1 predicting response bias (*c*) on the Mooney face detection task using age, sex, and recruitment group (Model 1) and *Repetitive Behaviours* subscale scores from the Comprehensive Autistic Trait Inventory (Model 2) as independent variables.

|  |  |  |  | **95% Confidence Interval** | |
| --- | --- | --- | --- | --- | --- |
|  | ***t*** | ***p*** | **β** | **Lower** | **Upper** |
| **Model 1: *F*(3, 316) = 0.85, *R^2^* < .01, p = .47** | | | | | |
| Constant | -1.265 | .207 |  |  |  |
| Age | 0.978 | .329 | 0.071 | -0.072 | 0.213 |
| Sex | 0.824 | .410 | 0.050 | -0.069 | 0.169 |
| Recruitment group | 0.450 | .653 | 0.033 | -0.111 | 0.177 |
|  |  |  |  |  |  |
| **Model 2: *F*(4, 315) = 1.34, *R^2^* = .02*, p* = .26; *ΔR^2^* = .01, *p* = .10** | | | | | |
| Constant | 0.041 | .967 |  |  |  |
| Age | 0.611 | .542 | 0.045 | -0.100 | 0.190 |
| Sex | 0.865 | .388 | 0.052 | -0.067 | 0.171 |
| Recruitment group | 0.558 | .577 | 0.041 | -0.103 | 0.184 |
| Repetitive behaviour | -1.669 | .096 | -0.096 | -0.209 | 0.017 |
